# The Field of Science

**Published:** 1893-12-30

**Authors:** 


### THE FIELD OF SCIENCE.

In the absence of any specialised information regarding tropical diseases, the question is being mooted in India of the feasibility of convening a medical congress next year, at some large centre, such as Calcutta or Madras. So far the project has not assumed any very definite shape, but the suggestion is a timely one, and we shall hope that it will soon be matured. It will readily be seen on all hands that a free discussion on the medical, surgical, and sanitary work of India would not only have a local importance, but would be a matter of a yet wider public interest to us all.

It is interesting for a moment to turn from the study of modern preventive hygiene in the West to the treatment of disease in the East, where its expulsion and dissemination takes the place of the sanitary precautions of a higher civilisation. Indeed, the mediate expulsion of ills through a material medium which is so widely adopted in the far East is the psychological antitheses of their immediate expulsion in the West through visible medical forms. With us in Europe the prevention of ills is of equal importance to their cure: among more primitive nations this is not the case. Here, instead, we find the propitiatory sacrifice, and the scape-goat principle carried out in varied forms. In certain of the Pacific Islands (to quote an instance or two in illustration of this), the disease is "exterminated" through the offices of a canoe, which, adorned with many flowers, bears away with it to sea the demon of the molesting disease.

In the Central Provinces of India an equally harmless ceremony takes place. Rice and a straw from the roof of each infected dwelling are burnt together at some appointed shrine to propitiate the evil spirits, and offered by the priests in the spirit of a sacrifice. Less harmless is the custom of the Bhars. Here, when cholera breaks out among these people, a female goat or buffalo is conducted in great state outside the boundary line, and not allowed to return. The disease thus "transferred" from one village to another continues to be passed on in the same manner from place to place. The spirit of this neighbourly act is characteristically embodied in the words, "Take away from here all kinds of sickness; take them to other islands, to other lands, which lie eastward, where the sun rises."

It is now finally decided that the memorial to Sir Richard Owen is to take the form of a full-length marble statue to be executed by Mr. Thomas Brock. This, when completed, will be placed in the South Kensington Museum.

"The National Congress for the free exercise of medicine," which met recently at Paris, was convened on a somewhat less "professional" footing than have been other therapeutical congresses so far. The speakers at this particular meeting, as indeed all those invited to take part in it, included such persons only as had given themselves up to the physical curing of mankind without being duly qualified members of the medical profession. A heterogeneous collection these, including amongst others faith-curers, masseuses, pastors, electricians, sisters of charity, and occultists. And not only were the exponents of medicine in its "untrammelled exercise" here represented, but also the "cures" themselves were allowed a hearing. A handful of persons whose "cases" were described as having failed to be reached through the more definitely accepted channels of medical assistance. If from its novelty alone, this congress no doubt had its attractive side, and we shall quite expect the quackery exhibition (shall we call it ?) to be repeated in other countries, after the precedent thus established.

The return of Professor Garner and his story of the sympathetic chimpanzees recalls the fact (remarks a contemporary) that he had the late Sir Richard Burton as his precursor in the serious study of the simian language. In fact, Lady Burton records in her recent biography how, growing weary of the manners and conversation of the regimental mess, her husband sought refreshment in the society of forty monkeys, who sat down to dinner with him daily, and were honoured with various official titles suited to the appearance and capacity of each. One of them, a pretty little silken creature, as she assures us, without a sign of jealousy, was called his wife, and had a lovely pair of pearl earrings. Burton declared that he had quite mastered the elements of their speech, and was able to keep up a tolerably fluent conversation with his loquacious friends. He made a list of some sixty of their most familiar words, but, unhappily, the valuable document was lost in a fire. Since those days Professor Garner has constituted himself interpreter of the chimpanzee language, into the nature of which he has recently made a very exhaustive study.

